# Migration timing and marine space use of an anadromous Arctic fish (Arctic Char, *Salvelinus alpinus*) revealed by local spatial statistics and network analysis

**DOI:** 10.1186/s40462-024-00455-z

**Published:** 2024-02-03

**Authors:** Rosie Smith, Eric Hitkolok, Tracey Loewen, Amanda Dumond, Heidi Swanson

**Affiliations:** 1 Department of Biology, University of Waterloo, Waterloo, ON Canada; 2 Kugluktuk Hunters and Trappers Organization, Kugluktuk, NU Canada; 3 Fisheries and Oceans Canada, Freshwater Institute, Winnipeg, MB Canada; 4 Department of Biology, Wilfrid Laurier University, Waterloo, ON Canada

**Keywords:** Acoustic telemetry, Arcti, Arctic Char, Dolly Varden, Getis, Local spatial statistics, Migration, Network analysis, Ocean, Summer

## Abstract

**Background:**

The ice-free season (typically late-June to early-October) is crucial for anadromous species of fish in the Arctic, including Arctic Char (*Salvelinus alpinus*), which must acquire adequate resources for growth, reproduction, and survival during a brief period of feeding in the marine environment. Arctic Char is an important food fish for Inuit communities across the Arctic. Understanding drivers and patterns of migration in the marine environment is thus essential for conservation and management of the species.

**Methods:**

We used passive acoustic telemetry to characterize migration patterns of 51 individual anadromous Arctic Char during the ice-free season in the marine environment of Coronation Gulf (Nunavut, Canada; 2019–2022). Based on recent genetic evidence, some tagged individuals were likely Dolly Varden (*Salvelinus malma malma*), a closely related species to Arctic Char. Using local Getis G* and network analysis, we described movement patterns and identified high-use locations in the marine environment. We also related freshwater overwintering location to migration timing and movement pattern.

**Results:**

Comparing groups of fish that overwintered in distinct locations, we found: (i) limited evidence that marine movements were associated with overwintering location; (ii) minor differences in use of marine space; and, (iii) timing of freshwater return differed significantly between overwintering groups, and was related to length and difficulty of the migratory pathway in freshwater. Results from both network analysis and local Getis G* revealed that, regardless of overwintering location, coastal locations were highly used by fish.

**Conclusions:**

Overwintering locations, and the migratory routes to access overwintering locations, affect the timing of freshwater return. Preference of fish for coastal marine locations is likely due to abundance of forage and patterns in break-up of sea ice. Similarities in marine space use and movement patterns present challenges for managing this and other mixed stock fisheries of anadromous *Salvelinus* spp. Absences or periods of time when fish were not detected prevented comprehensive assessment of movement patterns. Local Getis G*, a local indicator of spatial association, is a helpful tool in identifying locations associated with absences in acoustic telemetry arrays, and is a complementary method to network analysis.

**Supplementary Information:**

The online version contains supplementary material available at 10.1186/s40462-024-00455-z.

## Background

Migration is an important adaptive behaviour in response to fluctuations in resource availability and/or habitat suitability over space and time. Anadromy (movement from freshwater to the ocean to feed) is a common migration tactic among Arctic species of fish, and is thought to be a response to the low productivity of freshwater systems relative to marine systems [1]. Arctic Char (*Salvelinus alpinus*) is a facultatively anadromous salmonid with a Holarctic distribution [2–4]. Anadromous individuals take advantage of rich marine food sources during the brief (often < 3 months) ice-free season and return to freshwater before the ice-covered season due to low salinity tolerance at cold temperatures [5, 6]. Anadromous Arctic Char cease or reduce feeding overwinter [7–9], which results in substantial decreases in body mass and energy stores [10–12]. Brief, annual periods of marine foraging during the ice-free season are thus essential for growth and regaining body condition after losses during the ice-covered season, and have major implications for reproduction and survival.

Arctic Char is important for both commercial [e.g., 13] and subsistence [14] fisheries across the Canadian Arctic; Arctic Char is highly valued by Inuit communities and local harvesting has numerous social, economic, cultural, and nutritional benefits [15]. Due to plasticity in life history and migration patterns of Arctic Char, effective stewardship requires location-specific information. Among populations, anadromous individuals exhibit high variability in frequency and timing of marine migrations [e.g., 16, 17], as well as distance traveled to reach marine foraging grounds (25–126 km [18, 19]). Extensive stock mixing (multiple populations occurring in sympatry) in the marine environment has been observed in some regions [20, 21], whereas distinct, stock-specific marine migration routes have been observed in other regions [19].

The Coppermine River supports a critically important subsistence fishery for Arctic Char for the community of Kugluktuk, Nunavut (67° 48’ N, 115° 05’ W). Whereas anadromous Arctic Char typically overwinter in lakes across their range, they are known by Inuit fishers and more recently by scientists [22] to overwinter in the fluvial environment of the Coppermine River. High inter-individual and inter-annual (within individual) variability in overwintering location has been observed within the Coppermine River system, but Arctic Char that overwinter in different locations within the Coppermine River enter the marine environment of Coronation Gulf at a similar time [22]. The Coppermine River system thus provides a unique opportunity to study and compare the movement patterns of Arctic Char that: (i) exhibit plasticity in freshwater migratory pathway and overwintering destination; but, (ii) enter the marine environment from the same freshwater system at a similar time.

Acoustic telemetry is a proven tool for tracking of aquatic animals, and a growing number of studies employ acoustic telemetry in both freshwater and marine environments [23–25]. Historically, research on Arctic Char has focused on the freshwater or estuarine component of migration [e.g., 26–30], because individuals are easier to capture when moving through restricted migration corridors. Studies in marine environments have relied on extensive netting campaigns [e.g., 31] or tag returns from commercial fisheries [e.g., 32]. Acoustic telemetry has allowed researchers to gain insights into the movement and ecology of Arctic Char in the marine environment [e.g., 19, 20, 33–36], but there remain fundamental gaps in our knowledge of space use and movement patterns in remote and understudied Arctic marine environments. Network analysis is a useful tool for analyzing acoustic telemetry data from passive receiver arrays [37, 38]. Local indicators of spatial association, such as local Getis G_i_*(d) statistics (hereafter G*; [39]) have been applied to acoustic telemetry data [e.g., 40–42], but their use remains uncommon. Here, we used network analysis and local Getis G* to address four main objectives that were aimed at informing management of the subsistence Arctic Char fishery in Kugluktuk, NU, and guiding future research priorities: (i) Describe movement patterns of anadromous Arctic Char in the marine environment; (ii) Determine if movement patterns in the marine environment correspond with known variability in overwintering location [22]; (iii) Identify high-use locations for anadromous Arctic Char in the marine environment and qualitatively compare marine space use between overwintering groups; and, (iv) Identify locations within the array that were associated with absences or lack of detections.

This study was originally conceived based on the conventional knowledge that all anadromous *Salvelinus* fish east of the Mackenzie River are Arctic Char. Some limited older [43] and very recent [44] genetic evidence indicates, however, that some of the char that use the Coppermine River are Dolly Varden (*Salvelinus malma malma*). Some individuals tagged in this study may therefore be Dolly Varden rather than Arctic Char. Dolly Varden are closely related to Arctic Char and, together with Bull Trout (*Salvelinus confluentus*), have been considered a “species complex” [see 45]. Like Arctic Char, Dolly Varden are facultatively anadromous, iteroparous, and intermittent spawners [46–48]. Dolly Varden have been documented occurring in sympatry with Arctic Char elsewhere [e.g., 49], but visual distinction of the two species is complicated by the fact that phenotypes of both species are highly variable [e.g., 50, 51]. We refer to tagged individuals as Arctic Char in the Methods and Results, but consider the findings in relation to presence of both species and uncertainty in species composition in the Discussion.

## Methods

### Study location

**Fig. 1:**
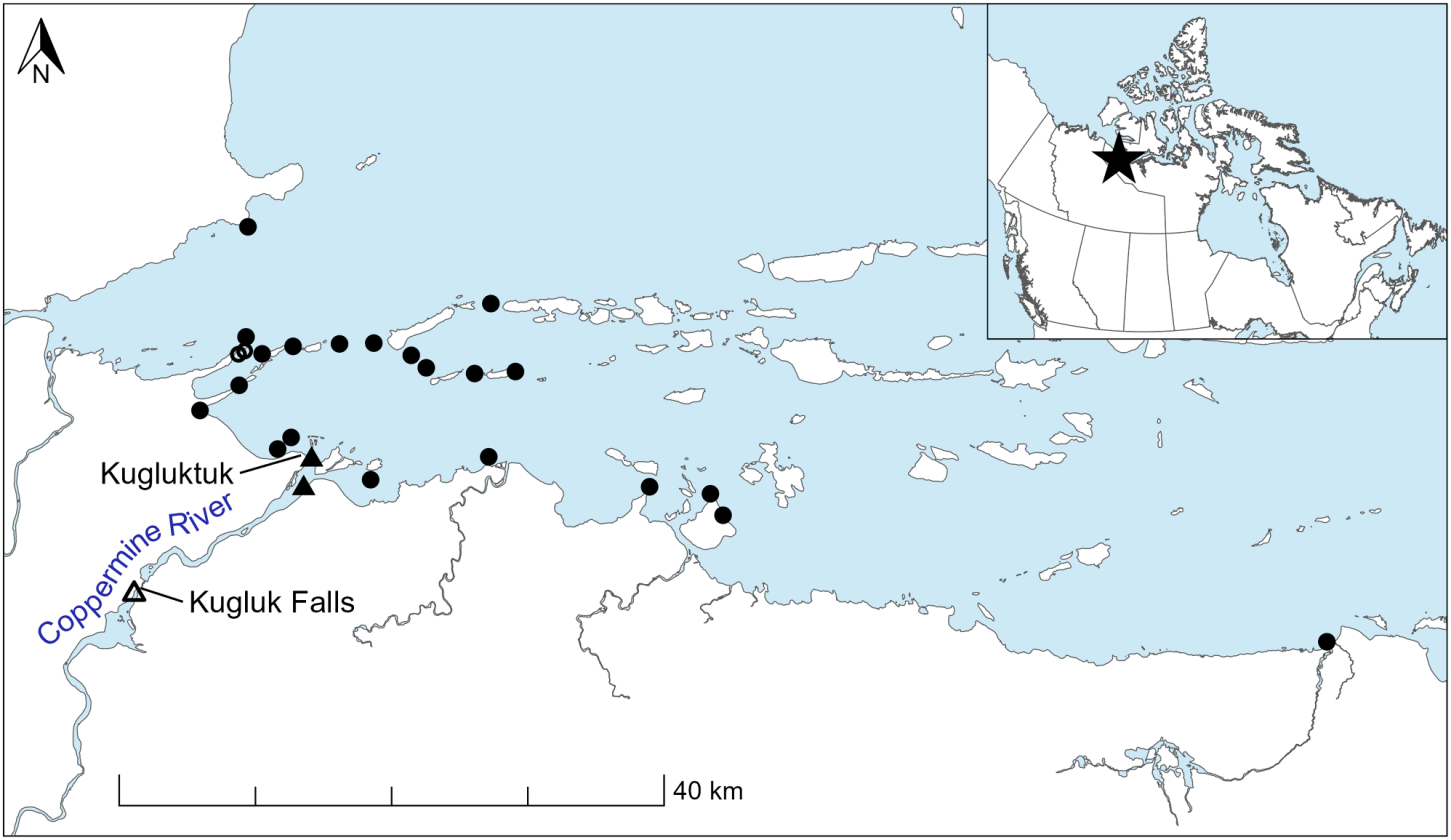
Map of the study area and locations of acoustic receivers that were consistently deployed during the ice-free seasons in all study years (2019–2022). Filled circles indicate marine receivers that were used for both network and local spatial (Getis G*) analyses. Open circles indicate marine receivers that were used individually to calculate G* and combined to generate networks. Filled triangles indicate receivers located at the mouth of the Coppermine River, which were used to identify timing of return to freshwater. Open triangle indicates a receiver located above Kugluk Falls, which was used to identify individuals that overwintered above the falls

This study was conducted in the marine environment of Coronation Gulf, located in the western Kitikmeot region of Nunavut, Canada (Fig. 1). Numerous islands are present in the area, resulting in complex bathymetry. Few bathymetric measurements are available for Coronation Gulf [52]; the maximum depth that was opportunistically recorded during this study was 90 m. Coronation Gulf has relatively low salinity, reaching a maximum of ~ 29 PSU [53]. Over a thirty-year period (1991–2020), the typical timing of break-up of sea ice in Coronation Gulf was 16 July and the typical date of freeze-up of sea ice was 29 October [54], resulting in a typical ice-free season of fifteen weeks. Local observations indicate that the timing of both break-up and freeze-up is increasingly variable and, in general, break-up is occurring earlier and freeze-up is occurring later (A. Dumond and E. Hitkolok, pers. obs.).

The Coppermine River flows into Coronation Gulf directly to the east of the Hamlet of Kugluktuk, Nunavut (Fig. 1). The Coppermine River provides a year-round source of freshwater to Coronation Gulf; mean discharge during the ice-free season is 473 m^3^/s and mean discharge during the ice-covered season is 118 m^3^/s [55]. Break-up and freeze-up dates for river ice are earlier than those for sea ice. The dates of river break- and freeze-up during the study period from 2019 to 2022 were determined from time-lapse cameras (Kugluktuk Ikaarvik youth group, unpublished data) and personal observations by the authors. Break-up of river ice ranged from 15 to 21 June, and freeze-up ranged from 01 to 30 October. Kugluk Falls, which poses a challenging but passable obstacle for migrating Arctic Char, is located approximately 17 km upstream of the mouth of the Coppermine River (Fig. 1).

### Acoustic tagging and receivers

Detailed methods for capture and tagging of Arctic Char are available in Smith et al. [22]. Briefly, Vemco V16T acoustic tags (InnovaSea, Halifax, NS) were surgically implanted into 198 adult Arctic Char that were captured in Coronation Gulf or the Coppermine River in 2018 (*n* = 48), 2019 (*n* = 117), and 2021 (*n* = 33). All fish selected for tagging had no evidence of injuries, disease, cysts, or deformities, and had reasonable body condition, based on local knowledge of typical body condition for the time of year that tagging occurred. Tags transmitted fish ID at random intervals, with a mean interval of 90 s.

An array of acoustic receivers was deployed year-round in the Coppermine River and Coronation Gulf. Receivers were deployed along migration corridors identified by local Inuit fishers and along coastal features where it was suspected that fish would pass while traveling through the study area. Complete datasets are available from twenty-three receivers in the marine environment, two receivers at the river mouth, and one receiver above Kugluk Falls during four ice-free seasons from 2019 to 2022 (Fig. 1). Receivers were deployed ~ 1–1.5 m above the river or ocean bottom and at locations where water depths ranged from 2.8 to 60 m.

### Detection data

Detection data were downloaded from receivers at least once per year and uploaded into VUE 2.8.1 (InnovaSea, Halifax, NS). The VRL File Editor in VUE was used to adjust recorded times to account for drift of receiver clocks. All further data manipulation and analyses were conducted in R version 4.2.2 [56], including manual correction of clock times that could not be corrected in VUE. False detections (invalid tag IDs resulting from incomplete transmissions or interference among transmitter signals) were removed from the dataset. To account for potential invalid detections with valid tag IDs that may result from signal collisions, single detections and rapid transitions between receivers were manually reviewed for feasibility. Detection data were condensed into residence events, which were defined as a period of time that an individual fish was detected by a single receiver with no gaps in detection greater than 24 h. Fish were observed moving only to neighbouring receivers (< 6000 m) in 97% of instances when fish moved away from, and then returned to, the same receiver within a 24-hour period. We thus considered a threshold of 24 h acceptable to define residence events. Detections from potential mortalities or expelled tags were removed following Smith et al. [22] before subsequent analyses.

### Overwintering location

Fish were assigned to one of two overwintering locations: (i) above Kugluk Falls, if they were detected at the receiver located above the falls (Fig. 1) and were not detected by other receivers until the following spring; or, (ii) below Kugluk Falls, if they were detected during the ice-covered season at one or more receivers deployed in the Coppermine River below the falls. All data analyses were restricted to fish with a complete record of marine migration in a given year (i.e., fish were tagged in a previous year and were not flagged as potential mortalities or expelled tags in the year in question), more than one marine residence event, an identified date of freshwater return (see below), and an identified overwintering location (above or below Kugluk Falls).

### Movement patterns in the marine environment

#### Return to freshwater

The date that each fish entered freshwater following marine migration was identified as the first detection at a receiver at the mouth of the Coppermine River (Fig. 1), provided all subsequent detections in that year were at river receivers. If multiple consecutive residence events (i.e., detection periods separated by more than 24 h) were observed at the river mouth, potentially representing fish approaching but not entering the river, the residence event with the latest date was used as the date of freshwater return.

#### Individual network metrics

Network analysis was used to characterize the movement patterns of individual fish through the study area. Individual networks were constructed for each fish and year. Receiver locations were represented by network nodes and movements of individuals among receivers were represented by network edges. Networks were undirected (did not consider direction of travel). Two marine receivers were located 530 m apart and often had overlapping detection ranges (i.e., an individual tag transmission was recorded by both receivers). To prevent overlapping detections from generating an inordinate number of movements for network analysis, the detections from these two receivers were combined to form a single node. Twenty-three nodes were included in network analyses, including twenty-one nodes representing individual marine receivers deployed in all study years (2019–2022), one node representing the two combined marine receivers, and one node representing two combined receivers at the mouth of the Coppermine River (Fig. 1). The river node was included to capture movements of fish that briefly entered the river before returning to the marine environment within a single ice-free season.

For each individual fish in a given year, four global or whole-network metrics were calculated to characterize movement patterns and describe network structure: node density, edge density, network diameter, and clustering coefficient. These network metrics were selected for their relevance to Arctic Char movement patterns and each described a different aspect of movement in the marine environment (Table 1). Node density was calculated as the proportion of nodes used, and represented the proportion of the network used by an individual [57]. Edge density, the proportion of edges used, represented the mobility of an individual [57], and was calculated using the *edge_density* function in R package *igraph* [58]. Although edge density is likely to be lower for fish that occupy areas of the network where receivers are located farther apart, edge density was included because (i) all fish enter and exit the marine environment from the same location at the mouth of the Coppermine River and therefore have equal opportunity to move throughout the network; and, (ii) considering edge density in conjunction with other metrics such as Furthest distance (Table 1; see below) can reveal differences in movement patterns. Network diameter was determined by calculating the shortest paths (along observed edges) between all pairs of observed nodes, and identifying the longest of these shortest paths [59]. Network diameter thus provided a distance measure of the spatial extent of the network used by an individual. Network diameter was calculated using the *diameter* function in *igraph*. For the calculation, distances between pairs of nodes were used as edge weights. As the coastline of the study area is highly variable with numerous islands, distances measured along observed edges sometimes crossed land or the detection radii of receivers that had not detected the fish in question. To adjust distance measurements to avoid land and other receivers, shapefiles of waterbodies from the CanVec hydrographic series [60] were imported into R and a buffer of 500 m (the approximate detection radii of receivers) was calculated around each receiver location using the *st_buffer* function in R package *sf* [61]. For each pair of receivers, a raster with approximately 10 m x 10 m cells was generated with conductance values of NA (Not Applicable; movement not possible) for raster cells on land or within the buffer of other receivers (i.e., receivers other than the pair in question), and conductance values of 1 for raster cells in water and outside the range of other receivers. Minimum within-water distances were then calculated using the R package *gdistance* [62] with 16-cell neighbourhoods. Finally, the clustering coefficient was calculated as the probability that two neighbours of a given node were themselves connected by an edge [59]. Fish that have a tendency to circulate within a region or regions, rather than make directed movements through the study area, would have high clustering coefficients. The clustering coefficient of each individual network was calculated using the *transitivity* function in *igraph*. A summary of metrics is provided in Table 1.

#### Distance traveled

Minimum total distance traveled (Total distance; Table 1) was calculated as the sum of the distance from the mouth of the Coppermine River to the first marine receiver where an individual was detected, the distances between receivers for all observed marine movements, and the distance from the last marine receiver where an individual was detected back to the mouth of the Coppermine River. Distance of furthest observation (Furthest distance; Table 1) was identified as the furthest distance from the Coppermine River to a receiver where an individual was detected. All distances were calculated in water (i.e., paths did not cross land features), as outlined above.

**Table 1:** Summary of metrics used to describe individual movement patterns and identify high-use locations in the marine environment. Global metrics reflect movement patterns of individual fish in a given year. Local metrics describe space use at a given location (node, for network analysis; receiver, for G*). Italicized metric names indicate metrics that were calculated using network analysis

	Description	Indicates
**Global metrics (individual fish)**		
*Node density*	Proportion of nodes used out of all possible nodes	Proportion of the network used by an individual
*Edge density*	Proportion of edges used out of all possible edges	Mobility of an individual
*Network diameter*	Longest of shortest paths along observed edges between all pairs of observed nodes	Spatial extent of the network used by an individual
*Clustering coefficient*	Probability that two neighbours of a given node are themselves connected by an edge	Circulation within a region (high clustering coefficient) or directed movements (low clustering coefficient)
Total distance	Minimum total distance, from Coppermine River, along observed edges, back to Coppermine River	Minimum observed mobility of an individual
Furthest distance	Furthest distance from the Coppermine River to a receiver where an individual was detected	Minimum furthest distance traveled by an individual
Days undetected	Proportion of days during the ice-free season (until freshwater return) that an individual was not detected	Absence from array or movement outside detection range of receivers
Freshwater return	Decimal day of year when an individual returned to freshwater (the Coppermine River) to overwinter	Time that marine foraging ceased
**Local metrics (receiver locations)**		
*Node strength*	Number of movements to and from a given node, including self-edges	Locations with high activity (number of movements)
*Restricted betweenness*	Number of movements that pass through a given node	Movement corridors
Local Getis G*	Duration of residence events within 6000 m^1^of a given receiver, relative to time spent at all locations	Regions with high activity (duration of residence events)

^1^A distance of 6000 m was selected to ensure all receivers had at least one neighbour, with the exception of four receivers that had no similar neighbours

#### Days undetected

Estimates for network and distance metrics may be biased due to the configuration and coverage of the network. For example, for individuals that occupy areas outside of the detection radii of network receivers, estimates of node density, edge density, and minimum total distance will likely be lower. To investigate whether observed movement patterns were associated with absences or lack of detections within the array, we calculated the proportion of days that each individual was not detected during the ice-free season (beginning with river break-up and ending with freshwater return). Day was selected as the unit of time, as 24 h was used as the gap threshold for differentiating separate residence events (see above).

#### Cluster analysis

To determine if distinct movement patterns in the marine environment could be identified, cluster analysis was performed on the global metrics calculated for each fish (four network metrics (node density, edge density, diameter, clustering coefficient), total distance, furthest distance, days undetected, and freshwater return; Table 1). Clustering was conducted and validated using the *clValid* function in the R package *clValid* [63] with four clustering methods (hierarchical complete linkage agglomerative, hierarchical divisive, *K*-means, *K*-centroid), 2–6 clusters, and both internal and stability validation measures. Silhouette analysis, the gap statistic, and the elbow method were implemented using the *fviz_nbclust* function in the R package *factoextra* [64] to identify the optimal number of clusters. Clustering was based on Euclidean distances among scaled and centred variables. Clustering methods were ranked using the *RankAggreg* function in the R package *RankAggreg* [65]. Assigned clusters were compared for individual fish among years to determine if fish consistently displayed the same pattern. To visualize results of the cluster analysis, principal components analysis (PCA) was conducted using all variables that were used to identify clusters (eight global metrics; Table 1).

#### Movement patterns and overwintering location

To investigate whether there were differences in movement patterns between overwintering locations (above or below falls) and among years, two-way fully-crossed ANOVAs were used to compare global network metrics, minimum distances traveled, and freshwater return (Table 1) among overwintering locations and years (2019–2021). The year 2022 was excluded from this analysis, as no fish were detected overwintering below Kugluk Falls in 2022. When a fish met the criteria for inclusion in more than one year, only one year was included in ANOVAs to avoid dependence issues due to repeated measures. Years when the overwintering location was below Kugluk Falls were preferentially included, as well as records from 2021, to improve balance in sample sizes among groups. The final sample size for ANOVAs was forty-nine individuals. Levene’s tests were used to assess homogeneity of variance and Shapiro-Wilk tests were used to assess normality of residuals from the models. Edge density, diameter, furthest distance, and clustering coefficient were log-transformed to better meet the assumption of normality. Significant differences (*p* ≤ 0.05) among groups were investigated using post hoc Tukey HSD tests.

While ANOVAs allowed us to directly compare metrics among overwintering locations and years, linear mixed models allowed the full dataset to be modeled, including all years and repeated measures. For this reason, mixed models were used to relate global network metrics, minimum distances traveled, and overwintering location (categorical) to date of freshwater return (Table 1). All explanatory variables were scaled and centred. Multi-collinearity among variables was assessed using the function *vif* in the R package *car* [66]. After removing edge density and minimum total distance travelled, all variance inflation factors were < 3 (maximum 2.9; [67]). Because the study spanned multiple years and marine conditions were expected to vary among years, year was included as a categorical random factor. Fish ID was also included as a random factor in this analysis, because eighteen fish were detected in multiple study years. Following Zuur et al. [68], the global model (all fixed factors) was fit twice, using restricted maximum likelihood estimation, to identify the appropriate random structure with: (i) both random factors; and, (ii) fish ID only. Likelihood ratio tests were used to compare models, with the p value adjusted to account for testing on the boundary [68]. The model with both fish ID and year as random factors was significantly better than the model with fish ID as a random factor (*p* < 0.001). Therefore, both random factors were retained. All possible models, including a null model (random factors only), were fit using maximum likelihood estimation and compared using AICc. Generalized linear models were implemented in the R package *lme4* [69]. AICc, conditional R^2^, and marginal R^2^ were computed in the R package *MuMIn* [70].

### High-use locations and space use in the marine environment

Local (location-based) metrics were used to identify high-use locations for Arctic Char in the marine environment, and to compare use of space between overwintering groups. Two local network metrics were calculated for individual fish: node strength and restricted betweenness (Table 1). Local network metrics for individual fish were first standardized for inter-individual and inter-annual differences in freshwater return by dividing by the number of ice-free days before each individual returned to freshwater. Mean metrics were then calculated for each overwintering location (above or below Kugluk Falls).

Node strength was calculated as the number of movements both to and from a given node. Node strength included self-edges (consecutive residence events at the same receiver, separated by an interval greater than 24 h). Self-edges indicate repeated use of an area [71] and were included to reflect the importance of frequently used locations. Network configuration can bias node strength, as fewer movements are likely to be observed to/from nodes that are farther from neighbouring nodes than to/from nodes that are closer to neighbouring nodes. Self-edges are more likely to occur at nodes that are farther from neighbouring nodes and can be considered as movements to/from an unknown node. Thus, self-edges were included to help mediate bias due to network configuration. Node strength was calculated using the *strength* function in *igraph*.

Betweenness in weighted networks is a measure of the flow (in this case, movement) along the shortest path between each pair of nodes that depends on passing through a given node [72]. A modified definition of betweenness, used by Lea et al. [57], is the number of movements that pass through a given node (i.e., two consecutive edges, where the start and end nodes are different). We used the modified definition of betweenness, hereafter called restricted betweenness, because paths are restricted to two adjacent edges connecting three nodes. Restricted betweenness was calculated by identifying time series of residence events for each fish, where three consecutive residence events were at different receivers (i.e., no self-edges or movements back and forth between two receivers). High values of restricted betweenness indicated nodes that were used as movement corridors.

Both local network metrics (node strength, restricted betweenness) were calculated using observed movements, and did not consider length of time spent at each receiver location. Local Getis G* was used to identify hotspots (clusters of high activity) and coldspots (clusters of low activity) in terms of time, rather than movement. As a local measure of spatial association, G* relates the values within a distance, d, of a location relative to all values within the study area [73]. The durations of residence events at each receiver were summed and used to calculate G* values. To verify that our threshold for identifying residence events (24 h) did not impact G* results, we conducted the analysis using a range of thresholds (1–48 h; results are presented in Supplementary Information). Single detections were assigned a residence duration of 90 s (the mean transmission interval of the acoustic tags). To account for differences in the length of time receivers were deployed, the duration of residence events for each individual was scaled by the amount of overlap between receiver deployment and ice-free period (before freshwater return) in a given year. To account for differences in time of freshwater return, the duration of residence events for each individual at each receiver was also standardized by time from river break-up until freshwater return. The value chosen for d was 6000 m, which ensured that all receiver locations would have at least one neighbour, with the exception of four locations that had no neighbours that were similar in depth and coastal features. Similar to the distances used in network analysis, distances were calculated in *gdistance* to avoid land, but without the constraint of avoiding detection radii of other receivers. G* was calculated using the function *localG_perm* in R package *spdep* [74]. Weights were binary (1 for receivers within 6000 m of receiver *i*, 0 for receivers further than 6000 m from receiver *i*).

The three local metrics (node strength, restricted betweenness, G*; Table 1) were compared qualitatively between overwintering locations (above or below falls). High-use locations were identified by qualitatively comparing relative ranks of all three local spatial metrics. The receivers at the mouth of the Coppermine River were excluded, as they were not included in G* calculations. As discussed above, two marine receivers had individual G* values, but were combined to form one node for network analysis. To allow comparison of ranks among local metrics, the mean G* value of these two marine receivers was calculated and used as a single location in comparisons. Locations were assigned a rank for each spatial metric, and the three ranks were summed for each location to form a combined rank.

### Locations associated with absences

To identify locations associated with absences or lack of detections within the array, we counted the number of ice-free days (before freshwater return) that each fish was not detected either immediately preceding or immediately following detection at each receiver. Similar to the global metric of days undetected (see above), day was selected as the unit of time because 24 h was used as the gap threshold for differentiating separate residence events. The number of undetected days associated with each receiver was standardized for differences in timing of freshwater return by dividing by the number of ice-free days before each individual returned to freshwater. The mean standardized values for each receiver were used to calculate G* values using the same method as above. Statistical significance of G* values was determined using 1000 conditional permutations (holding the value at the central location constant while shuffling remaining values without replacement), using the function *localG_perm* in R package *spdep* [74] and following Ord and Getis [39].

### Mapping and data visualization

Data visualization was conducted using the R packages *ggplot2* [75] and *ggalluvial* [76]. Networks were visualized with R package *ggraph* [77]. Shapefiles of waterbodies were obtained from the CanVec hydrographic series [60]. Maps were arranged with R package *patchwork* [78] and annotated using R packages *ggspatial* [79] and *geomtextpath* [80].

## Results

Fifty-one fish met all four criteria to be included in analyses (complete record of marine migration, more than one marine residence event, identified date of freshwater return, and identified overwintering location) in at least one year of the study (2019–2022). Eighteen fish met the criteria in more than one year, yielding a sample size of seventy-three unique fish*year records. At the time of tagging, fork lengths of the fifty-one fish ranged from 539 to 857 mm, and weights ranged from 1850 to 5140 g. Tag burden was less than 1.8% of fish body weight. There were 34 343 detections recorded at the twenty-three marine receivers that were deployed consistently over the four years of the study (Fig. 1).

### Movement patterns in the marine environment

#### Return to freshwater

**Fig. 2:**
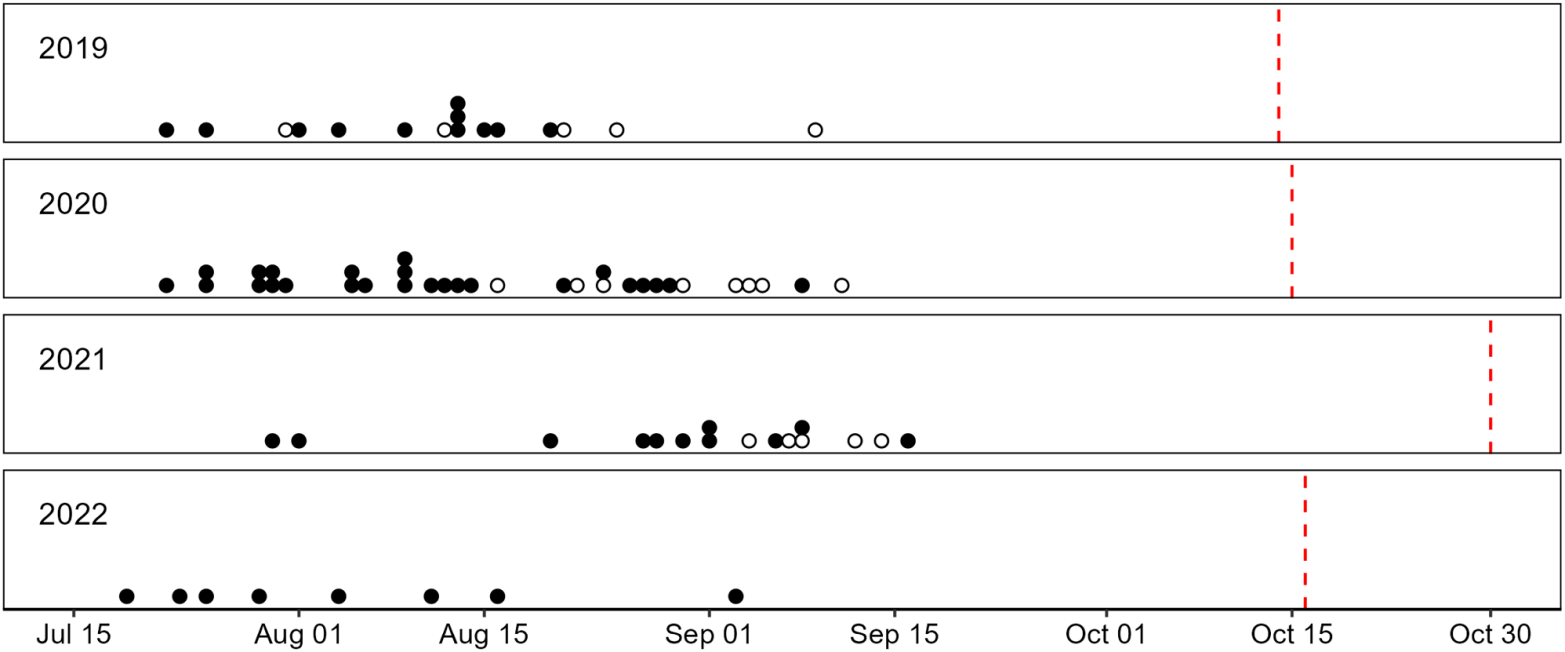
Dates of return to freshwater for fish that overwintered above (filled circles) and below (open circles) Kugluk Falls. Red dashed lines indicate the dates of freeze-up in the Coppermine River. Break-up dates for river ice were 19 June 2019, 21 June 2020, 18 June 2021, and 15 June 2022

The date of fall return to freshwater was highly variable among individuals and years. Date of freshwater return ranged from 18 July to 15 September (Fig. 2). Median date of freshwater return was 14 August.

#### Individual network metrics and minimum distances traveled

Metrics for individual networks were highly variable. Node density of the seventy-three records ranged from 0.04 to 0.70 (median = 0.35), or 1 to 16 (median = 8) of 23 possible nodes. Edge density ranged from 0 to 0.11 (median = 0.03), or 0 to 29 (median = 8) of 253 possible undirected edges, excluding self-edges. Diameter of observed networks ranged from 0 to 179.1 km (median = 55.8 km). Clustering coefficient ranged from 0 to 0.6 and was highly skewed (median = 0). The furthest distance that individuals were detected from the river mouth ranged from 13.6 to 78.8 km (median = 33.0 km). Approximately 40% of all individuals*years (*n* = 29) were detected at least once at the receiver located furthest from the mouth of the Coppermine River (i.e., 78.8 km). The total minimum distance that individuals were detected traveling in a single ice-free period ranged from 28.5 to 394.8 km (median = 162.9 km).

#### Cluster analysis

**Fig. 3:**
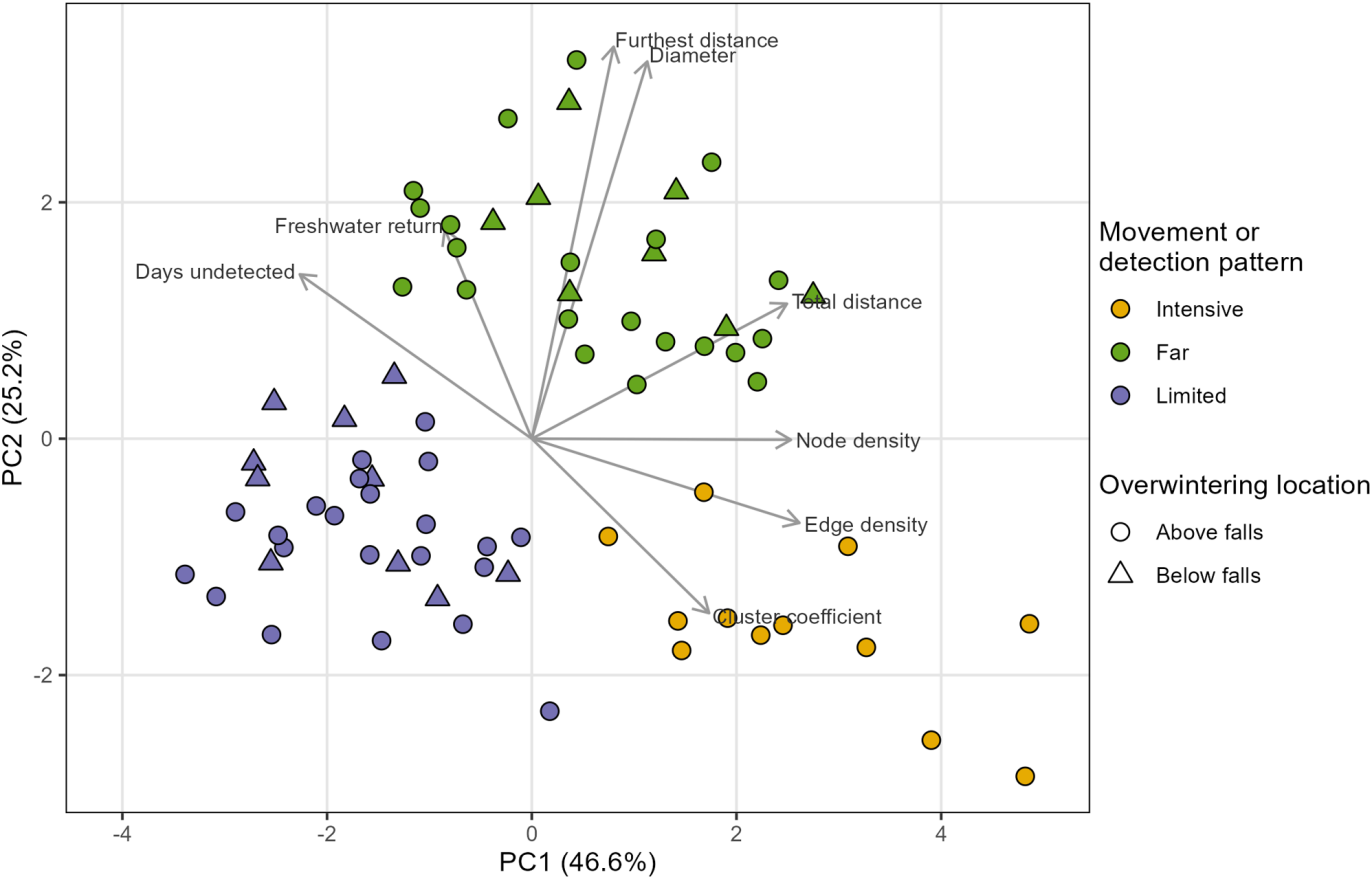
Principal components analysis of date of freshwater return (Freshwater return), proportion of days undetected (Days undetected), minimum total detected distance travelled (Total distance), distance of furthest detection (Furthest distance), and network metrics (Node density, Edge density, Diameter, Cluster coefficient; Table 1). Only the first two axes are displayed, as they explained a cumulative percentage of 71.8% of the variance and subsequent axes explained 12.3% or less of the variance. Colour indicates the movement or detection pattern observed, as inferred from *K*-means cluster analysis with three groups. Shape indicates overwintering location

Silhouette analysis, the gap statistic, and the elbow method consistently indicated the optimal number of clusters was three. Ranking of internal and stability validation measures indicated that *K*-means clustering with three groups was the most appropriate clustering method. The first two axes of a PCA, which included node density, edge density, diameter, cluster coefficient, total distance, furthest distance, days undetected, and freshwater return (Table 1), explained 72% of the variation in the data (Fig. 3). Three movement or detection patterns were identified when PCA results were compared with the *K-*means cluster analysis (with three clusters): intensive, far, and limited.

**Fig. 4:**
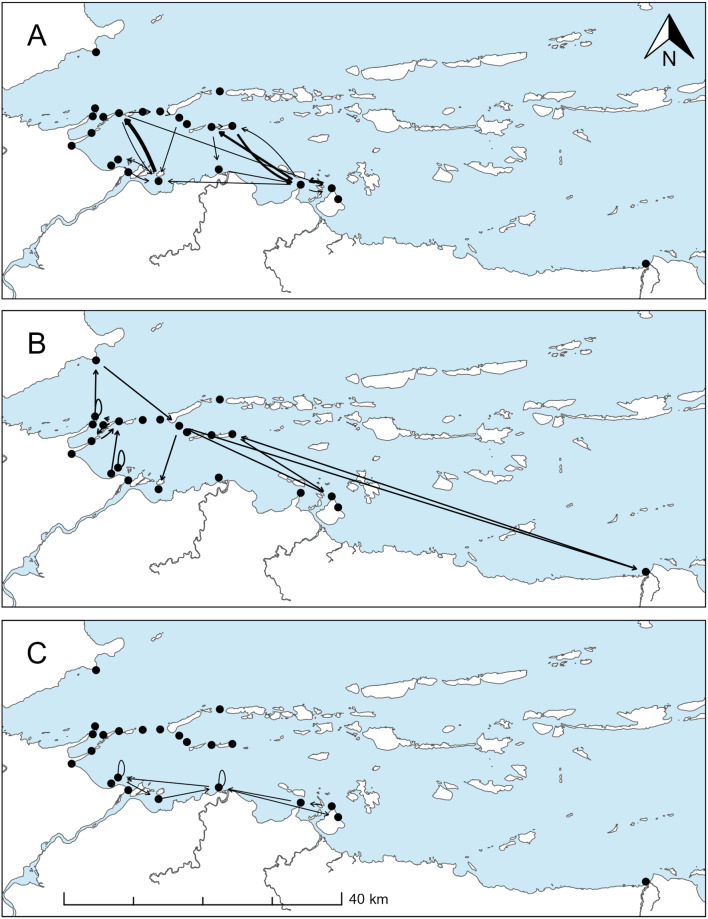
Examples of networks generated for the three observed movement or detection patterns, as identified by cluster analyses: (**A**) Intensive; (**B**) Far; and, (**C**) Limited. Network nodes (receivers) are indicated by black points. Edge weights (i.e., number of movements) are indicated by line thickness. Note that network edges are drawn for illustration purposes only; lines do not represent paths and lengths do not represent minimum distances traveled. Self-edges are indicated by loops that leave and return to the same receiver location

The intensive group was detected at locations relatively near the Coppermine River. Fish in this group had relatively early freshwater return and traveled intensively through a relatively restricted portion of the study area (high node and edge densities, high clustering coefficient, high minimum total observed distance; example network in Fig. 4a). The intensive group had a relatively low proportion of days undetected in the array (mean = 0.46, standard deviation (SD) = 0.20). Fish in the far group had the latest dates of freshwater return, were observed traveling relatively long distances, and were detected at the receivers furthest from the Coppermine River (example network in Fig. 4b). The far group had a relatively high proportion of days undetected in the array (mean = 0.77, SD = 0.10). Fish in the limited group had low observed node and edge densities, and were detected traveling short minimum distances (example network in Fig. 4c). Individuals in this group had the highest proportion of days where they were undetected in the array (mean = 0.86, SD = 0.09). Proportion of days undetected differed significantly among groups (ANOVA, F = 19.74, *p* < 0.0001, df = 2,46). Pairwise comparisons using Tukey HSD indicated that proportion of days undetected was significantly higher for the limited and far groups than for the intensive group (*p* < 0.0001), and that proportion of days undetected for the limited group was not significantly different from the far group (*p* = 0.26).

**Fig. 5:**
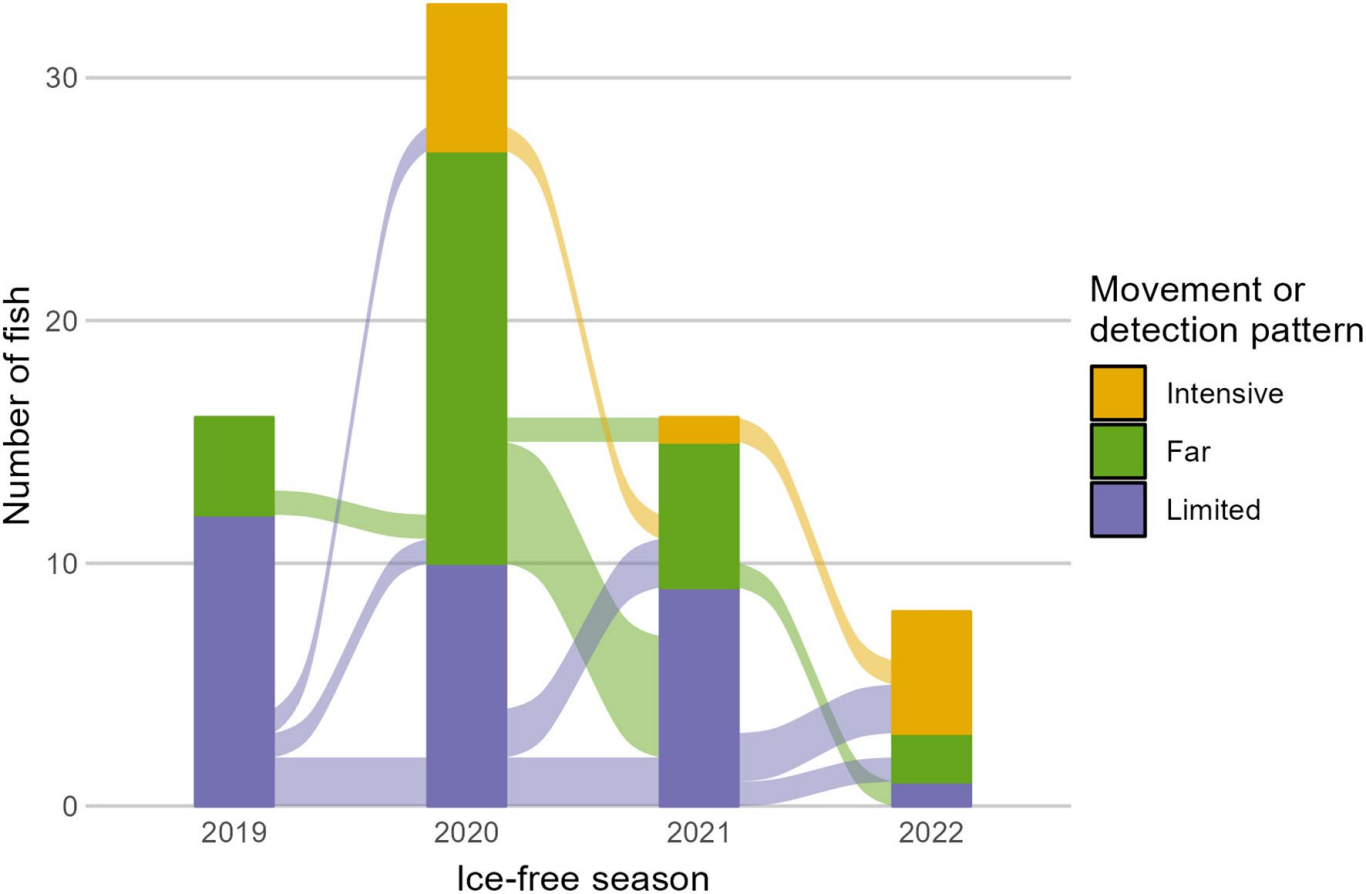
Observed movement or detection patterns of tagged fish in the marine environment during each ice-free season of the study. Colour connections between ice-free seasons indicate the pattern observed in the previous ice-free season (e.g., a yellow connection to a green bar represents an individual that exhibited the intensive pattern in one ice-free season and the far pattern in the following ice-free season)

All fish in the intensive movement group overwintered above Kugluk Falls (Fig. 3). There was no evidence that fish in the limited or far movement groups were associated with either overwintering location (above or below Kugluk Falls). For fish that were observed in more than one ice-free season (*n* = 18), movement or detection pattern was not consistent among years (Fig. 5).

#### Movement patterns and overwintering location

Date of freshwater return differed significantly both between overwintering locations and among years (ANOVA, F ≥ 11.103, *p* ≤ 0.00178, df = 2,43). The interaction between overwintering location and year was not significant (F = 0.097, *p* = 0.907). Pairwise comparisons using Tukey HSD indicated that freshwater return in 2021 was significantly later than freshwater return in both 2019 and 2020 (*p* < 0.0006), and that freshwater return in 2019 was not significantly different from freshwater return in 2020 (*p* = 0.51). Fish that overwintered above Kugluk Falls entered freshwater significantly earlier than fish that overwintered below Kugluk Falls (*p* = 0.0014). All other global metrics (Table 1) did not differ significantly between overwintering locations or among years (ANOVA, F ≤ 2.246, *p* ≥ 0.118, df = 2,43).

Overwintering location was the best predictor of date of freshwater return and explained 19.3% of the variation when included in a linear mixed model as the sole explanatory variable (with year and fish ID as random factors; Table 2). Global network metrics (describing individual movement; Table 1) and furthest detected distance were not good predictors of freshwater return and were ranked below or similar to the null model. When compared to the model with overwintering location alone, models that included both overwintering location and another explanatory variable had similar AICc scores, deviance (-2loglikelihood), and model fit (marginal R^2^, Table 2), indicating that other explanatory variables were noninformative [81].

**Table 2:** Subset of linear mixed models that related date of freshwater return to overwintering location, distance of furthest detection (furthest distance), node density, diameter, and clustering coefficient for fifty-one individuals. Eighteen individuals were observed in more than one year, for a total sample size of seventy-three observations. The null model includes only the random factors of year and fish ID. AICc scores were used to rank models, and models are presented in order of increasing AICc (decreasing rank). Marginal R^2^ values represent the proportion of variance explained by the fixed factors. Conditional R^2^ values represent the proportion of variance explained by both the fixed and random factors

Model	AICc	ΔAICc	-2log-likelihood	Marginal R^2^	Conditional R^2^
Overwintering location	591.19	0	580.30	0.193	0.416
Overwintering location + Furthest distance	591.98	0.79	578.71	0.206	0.452
Overwintering location + Node density	592.53	1.33	579.25	0.212	0.492
Overwintering location + Diameter	592.80	1.61	579.53	0.202	0.419
Overwintering location + Clustering coefficient	593.13	1.94	579.86	0.204	0.409
Overwintering location + Node density + Diameter + Clustering coefficient + Furthest distance	597.55	6.35	576.69	0.240	0.570
Furthest distance	608.54	17.34	597.64	0.026	0.357
Null	608.68	17.48	600.09	0	0.309
Node density	609.73	18.53	598.83	0.015	0.339
Diameter	610.21	19.02	599.32	0.008	0.321
Clustering coefficient	610.58	19.39	599.69	0.006	0.263

### High-use locations and space use

**Fig. 6:**
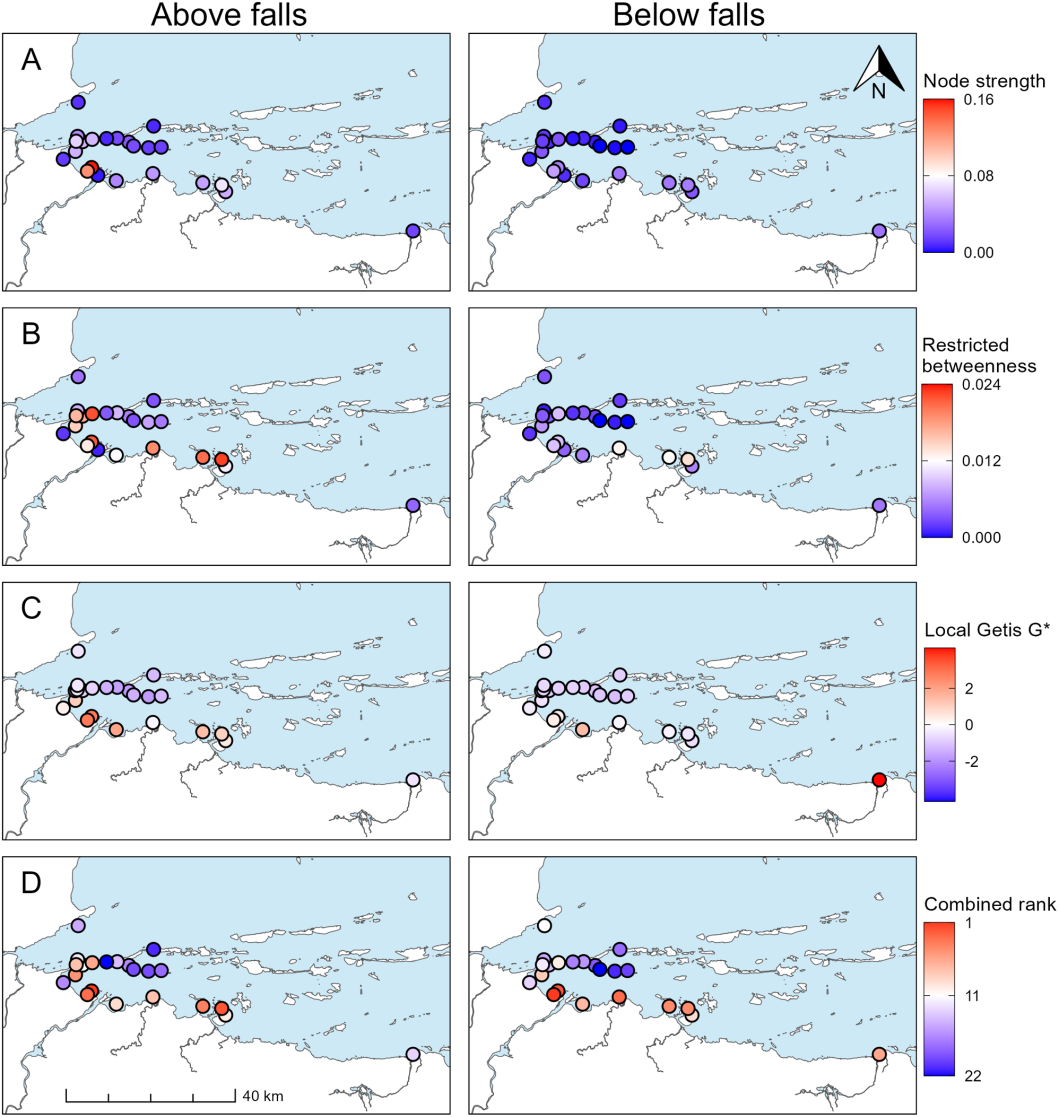
Values for local metrics: (**A**) Node strength; (**B**) Restricted betweenness; (**C**) Local Getis G*; and, (**D**) Combined rank for each receiver. For each measure, the scale is the same for fish that overwintered above Kugluk Falls (left panel) and those that overwintered below Kugluk Falls (right panel). Note that for local Getis G*, a value of 0 indicates that relative activity at that location was equal to the mean value for the whole study area. Similarly, negative values indicate locations where activity was observed, but relative activity was less than the mean value for the study area

Local metrics (node strength, restricted betweenness, G*; Table 1), which were used to indicate movement corridors and high-use locations, were generally similar between groups of fish that differed in overwintering location (above or below Kugluk Falls). Similar patterns in node strength were qualitatively observed between the two overwintering groups (Fig. 6a), except that fish that overwintered above Kugluk Falls made proportionally more movements to and from the receivers immediately to the west of the mouth of the Coppermine River. Restricted betweenness was generally similar between the two overwintering groups, although values at each receiver were consistently lower for fish that overwintered below Kugluk Falls (Fig. 6b). Higher values of restricted betweenness were typically observed at receivers located along the coastline to the east of the Coppermine River, receivers immediately to the west of the river mouth, and receivers located between the islands to the northwest of the river mouth, which indicated that these locations were important movement corridors. Qualitative assessment of local Getis G* values indicated that patterns in the relative time spent at locations across the study area were also largely similar between overwintering locations (Fig. 6c), except for two regions. First, G* values indicated the receivers immediately to the west of the mouth of the Coppermine River were greater hotspots for fish that overwintered above Kugluk Falls. Second, G* values indicated the receiver located furthest east in the study area was a greater hotspot for fish that overwintered below Kugluk Falls.

When receiver locations were ranked based on all three local spatial metrics, combined ranks were qualitatively similar between overwintering locations (Fig. 6d). Receivers that were located in deeper water (34–60 m), among offshore islands, typically had low combined ranks. Receivers that were located near the river mouth or along the coast typically had high combined ranks. The receiver with the greatest difference in combined rank between overwintering groups was the receiver located furthest east in the study. This location was ranked thirteenth for fish that overwintered above Kugluk Falls and sixth for fish that overwintered below Kugluk Falls.

### Locations associated with absences

**Fig. 7:**
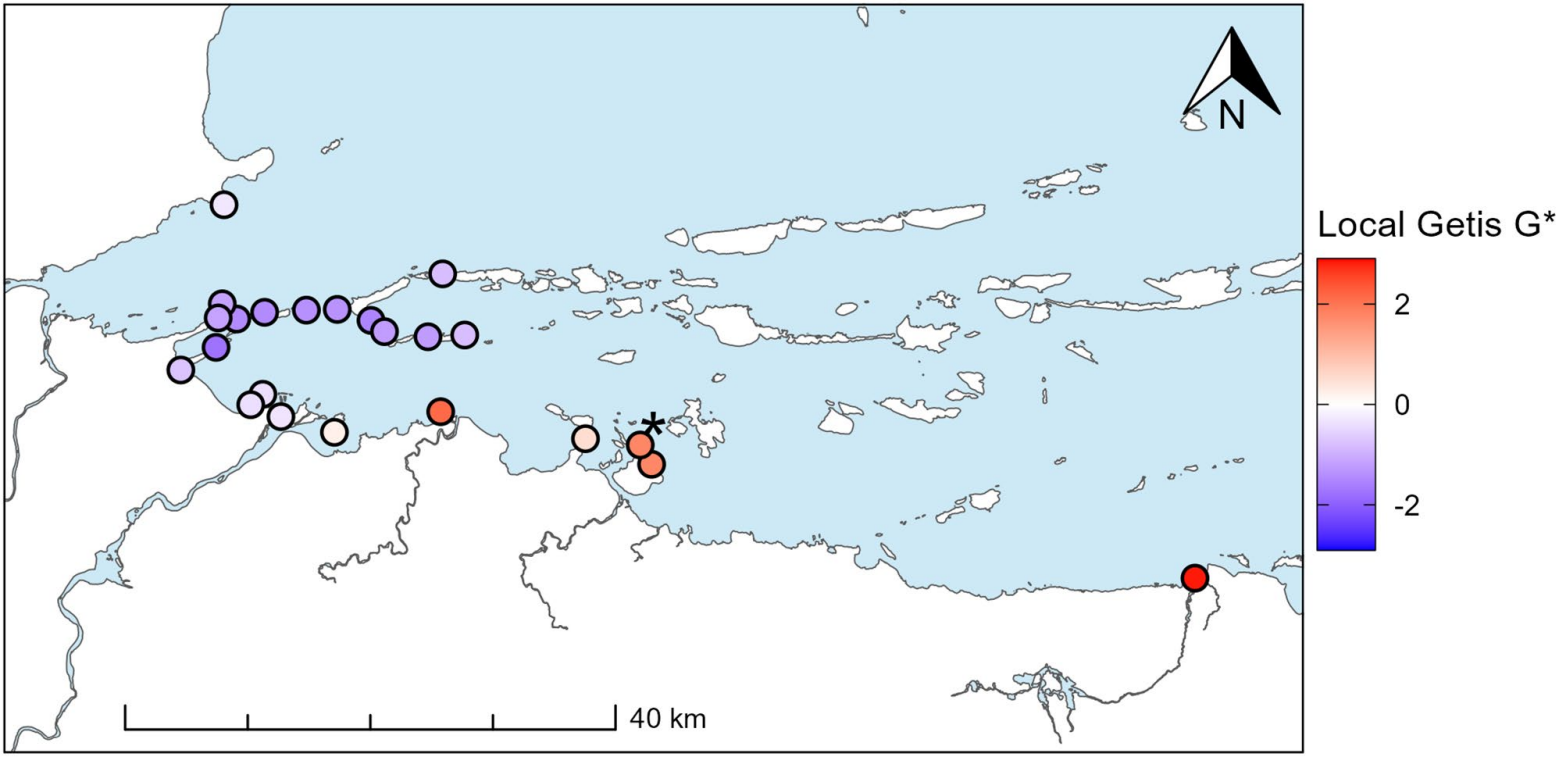
Local Getis G* of the number of undetected days associated with each receiver (i.e., days when a fish was not detected either immediately before arriving at or immediately after leaving a receiver). Star indicates a location with statistically significant spatial association (*p* < 0.05). Note that the receiver located furthest to the east had the highest G* value but did not have neighbours within 6000 m, so significance could not be assessed through conditional permutation

Qualitative assessment of local Getis G* values, a measure of local spatial association, indicated that receivers associated with the highest number of undetected days were typically located along the coast (Fig. 7). Conditional permutations indicated that spatial association was significant at one location, with a G* value of 1.77 (star in Fig. 7). The receiver located furthest east had the highest G* value (2.83; Fig. 7). This location did not have neighbours within 6000 m and, as conditional permutations keep the value at the central receiver constant and shuffle the remaining values, significance could not be assessed at this location.

## Discussion

### Movement patterns in the marine environment

Date of freshwater return was the only global (individual) metric that differed significantly among years. Freshwater return was highly variable among fish and years, and was significantly later in 2021 than 2019 or 2020; mean freshwater return was twenty days later in 2021 than in 2019 and seventeen days later in 2021 than in 2020. The dates of river freeze-up were respectively sixteen and fifteen days later in 2021 than in 2019 and 2020. Climate change is causing earlier break-up and later freeze-up of both river and sea ice throughout the Arctic [82, 83]. Changing ice conditions have impacted the migration patterns of both terrestrial [e.g., 84] and marine [e.g., 85] mammals, but similar impacts have not yet been observed in anadromous species of fish. Freeze-up likely does not directly affect timing of freshwater return in fall, because Arctic Char return to freshwater before freeze-up of either marine or river environments, but water temperature or other climatic factors that affect freeze-up may influence migration timing. Dempson and Kristofferson [32] found a negative relationship between mean catch date and sea temperature in commercial Arctic Char fisheries of Labrador, Canada. This suggests that water temperature influences migration timing, but environmental cues for freshwater return remain largely unknown for Arctic Char and Dolly Varden [but see 86]. Multi-year studies of multiple populations and geographic areas are necessary to understand migration cues and associated potential impacts of climate and other stressors.

Date of freshwater return was significantly later for fish that overwintered below Kugluk Falls than for fish that overwintered above Kugluk Falls. Inter-annual variability in overwintering location within individuals (i.e., the same fish overwintered in different locations in different years) has been observed in this system [22], which suggests that Arctic Char and Dolly Varden cannot be distinguished based on overwintering location (in relation to the falls). The difference in timing of freshwater return between overwintering groups is thus unlikely to be due to differences between Arctic Char and Dolly Varden in migration timing. Overwintering above Kugluk Falls involves a longer migration route and a challenging ascent. The length and difficulty of freshwater migration routes have previously been associated with life history type [87] and choice in overwintering location [88] in Arctic Char, but have not been investigated for Dolly Varden. This is, to our knowledge, the first evidence that suggests the length and/or difficulty of the migration route affects the timing of freshwater return for either species. Linear mixed models that accounted for inter-annual differences and repeated measures indicated that fish that overwintered above Kugluk Falls entered freshwater sixteen days earlier than those that overwintered below Kugluk Falls. As the ice-free period available for rich marine foraging is brief (often < 3 months), this represents a substantial reduction in the time available for growth and improvements in body condition for fish overwintering above Kugluk Falls, and may affect reproduction and survival.

We expected that individuals that overwintered above Kugluk Falls would not travel as far or as extensively in the marine environment as those that overwintered below Kugluk Falls, because individuals that overwintered above Kugluk Falls returned to freshwater earlier. We were generally unable to detect differences in movement metrics or patterns between overwintering groups, but all fish that exhibited the intensive movement pattern overwintered above Kugluk Falls. Fish in this group were detected making numerous movements, all relatively near the mouth of the Coppermine River. Movements over a relatively restricted area may be a tactic to conserve energy. While factors that affect overwintering locations used by fish within the Coppermine River system are unknown, there is no known spawning habitat below Kugluk Falls, and previous researchers have suggested that overwintering location may be related to spawning status or body condition [22]. Arctic Char and Dolly Varden are iteroparous and intermittent (i.e., not every year) spawners. Some anadromous Arctic Char [89] and Dolly Varden [48] are known to skip marine migrations altogether in years that they spawn, perhaps as a tactic to conserve energy. Restricting movement in marine environments during the ice-free season could allow fish to conserve energy while still allowing exploitation of rich marine food sources. Fish in the intensive movement group may thus conserve energy before ceasing marine foraging relatively early and completing the longer and more challenging migration above Kugluk Falls to spawn and/or overwinter. Further investigation of overwintering locations in the Coppermine River should include discrimination among locations at finer spatial scales, as well as assessments of fish spawning status and frequency.

The high proportion of days when individual fish were absent or not detected in the array suggests that the full extents of global (individual) metrics remain unobserved. The intensive movement or detection group had a significantly lower proportion of days undetected (mean = 0.46) than the far or limited groups, suggesting that the array configuration is appropriate and characterization of movement patterns is reasonable for fish in the intensive group. Fish in the far movement group were observed at receiver locations furthest from the Coppermine River, where receivers had few or distant neighbours. It is logical that individuals in this group had a higher proportion of days undetected, as there was less opportunity for detections during transition periods to and around these areas of the array. Although the full extent of movement for these individuals was unobserved, it is reasonable to conclude that their movements in the marine environment differed from the intensive group.

Fish in the limited group had the highest proportion of days undetected. Given this detection pattern and the array configuration, it is impossible to ascertain if individuals in this group demonstrated unique movement characteristics, or if movement was actually similar to either the intensive or far groups (but was unobserved). Additional detection information, likely involving an increase in number and locations of receivers, is required to adequately describe the movement patterns of this group of fish.

There are many logistical and environmental challenges associated with conducting aquatic research in remote areas, and decisions that balance effort and resources against scope and coverage must often be made with very limited or no prior information. In this understudied region, even limited information is valuable. Although the high proportion of days undetected for some individuals demonstrates the shortcomings of the array in terms of comprehensive or detailed assessment of movement patterns, the configuration and coverage of the array are not dissimilar from arrays in other studies that have attempted to describe marine migration of Arctic Char in the Canadian Arctic [19, 20, 33]. The locations of the receivers used in our analysis were consistent among study years, which allowed comparisons among study years. Fish entered and exited the marine environment from the same location and thus had equal opportunity to travel through (and be detected within) the study area each year, which allowed comparisons among individuals. While substantial uncertainty remains, particularly for fish in the limited group, movement or detection pattern was inconsistent among years for individuals with more than one complete year of summer movement data (i.e., the same individual exhibited different movement patterns in different years). This indicates that fish do not exhibit repeatable movement patterns or return to the same regions of the array each year.

The inter-annual variability observed within individuals suggests that movement was not uniquely related to innate factors, such as species, sex, or population. For example, because fish in the intensive movement group exhibited other movement groups in other years, we can infer that fish of the same species (Arctic Char or Dolly Varden) do not all exhibit the intensive movement pattern. There may yet be interspecific differences, such as the intensive movement pattern only being exhibited by one of the two species and with additional factors causing inter-annual variability in movement pattern, but this requires further investigation. The fork lengths of tagged individuals in our study were relatively large and within a relatively narrow range (539–857 mm), and results of relationships assessed between fish length and migration history (reconstructed from otolith microchemistry) for Arctic Char and Dolly Varden captured in the Coppermine River and nearby marine environment suggest that all tagged fish were repeat migrants (R. Smith, unpublished data). A comprehensive assessment of movement patterns and the drivers of inter-individual and inter-annual differences will require tracking both sexually immature and mature fish that reflect a wider range of fork length and migration experience, collection of additional information on spawning status and body condition, distinguishing between Arctic Char and Dolly Varden using new morphometric and genotypic information generated for the system [44], and greater receiver coverage in this remote and understudied region.

### High-use locations and space use in the marine environment

Although individuals that overwintered above Kugluk Falls left the marine environment earlier than individuals that overwintered below Kugluk Falls, there were similarities in patterns of space use in the marine environment between overwintering groups. This contrasts with the findings of Hollins et al. [19], who found that Arctic Char migrating to different overwintering lakes used distinct migration pathways and marine foraging areas. Stock mixing of Arctic Char during the ice-free season has been observed in the marine environment elsewhere in the Canadian Arctic [20, 32, 36] and there is some evidence that *Salvelinus* spp. from other river systems overwinter at least occasionally in the Coppermine River (R. Smith, unpublished data; A. Dumond and E. Hitkolok, pers. obs.). Also, as discussed above, some of the tagged individuals may be Dolly Varden rather than Arctic Char, and the two species cannot be differentiated based on overwintering location alone. It is thus possible that patterns of space use are similar between overwintering groups because overwintering groups are composed of both species. Genetic analyses to confirm the species of tagged fish is necessary to investigate if space use in the marine environment differs between Arctic Char and Dolly Varden, and if space use differs between overwintering groups within each species.

Although some marine locations, such as near the mouth of the Coppermine River, were consistently identified as high-use locations for both overwintering groups, local Getis G* revealed key differences in the proportion of time that each overwintering group spent at other locations. For example, individuals that overwintered below Kugluk Falls spent a greater proportion of time at the receiver located furthest to the east of the study area. Fish that overwintered above the falls also frequented the same eastern location, but spent proportionally more time at receivers near the mouth of the Coppermine River and at intermediate locations along the coast. The difference in time that the two overwintering groups spent at these locations may be due to differences in freshwater return and patterns in break-up of sea ice. Sea ice breaks up near the mouth of the Coppermine River soon after river ice (mid-June), and before breakup of sea ice along the coast (A. Dumond and E. Hitkolok, pers. obs.). Although Arctic Char have been observed under sea ice [22, 90, 91], extensive travel under sea ice is uncommon. It is likely that both overwintering groups remained near the Coppermine River and moved further from the river and along the coastline as the sea ice cleared. Individuals overwintering below Kugluk Falls returned to freshwater later, which allowed them to spend proportionally more time at distant locations before returning to freshwater.

Spatial patterns in break-up of sea ice may also explain which locations were highly used by anadromous Arctic Char and/or Dolly Varden. Local spatial metrics indicated that coastal locations were generally used more than locations offshore or near islands, in terms of both duration of activity (G*) and movement through the study area (node strength and restricted betweenness). Coastal receivers were relatively farther apart than offshore or island receivers, and both the number of movements associated with these receivers (affecting local network metrics) and the grouped duration of detections (G*) would be expected to be lower. Despite this spatial bias, estimates of local metrics were higher at coastal receivers. Our finding that coastal locations were highly used is consistent with previous research in Nunavut that showed that Arctic Char prefer nearshore environments [20]. Sea ice close to land breaks up earlier than sea ice further offshore (A. Dumond and E. Hitkolok, pers. obs.), and fish may spend more time and make more movements in areas that are ice-free earlier. Further, Capelin (*Mallotus villosus*), which are commonly preyed upon by Arctic Char in the marine environment [4], spawn on beaches in the Arctic [92] and are frequently observed along the coastline near Kugluktuk (A. Dumond and E. Hitkolok, pers. obs.). The preference for coastal areas that we observed may thus be a combination of environmental conditions and prey availability.

The true extent of offshore marine movement by Arctic Char has not been fully investigated. Although Arctic Char have been documented offshore (~ 10 km [20], ~ 25 km [32]), there were often very few or no receivers located offshore in previous studies that used acoustic telemetry [e.g., 20, 35, 36, 93]. To our knowledge, no studies have used satellite tags to obtain location estimates of Arctic Char. Based on the locations where pop-off satellite tags have detached, Dolly Varden have been observed frequenting offshore environments (2–152 km [94, 95]). A char was reported in the middle of Coronation Gulf (E. Hitkolok, pers. obs.), between Victoria Island and Kugluktuk, but it is unknown if the individual was an Arctic Char or a Dolly Varden. Although not ranked as high-use locations, tagged individuals were observed frequenting receiver locations near islands in Coronation Gulf. Complex bathymetry and lack of suitable environmental data make it challenging to determine if habitat use of tagged individuals near islands should be characterized as offshore or coastal. Further research is required to demonstrate both preference and full extent of movements of both Arctic Char and Dolly Varden in this and other areas.

### Locations associated with absences

Absences or periods when individuals are undetected are often ignored in acoustic telemetry studies [but see 96, 97], and may have important implications for data analysis and interpretation. In this study, a high proportion of days undetected precluded us from determining if limited or far movement/detection groups represented distinct movement patterns, and from fully characterizing movement in the marine environment. Resolving uncertainties surrounding absences is also important for identifying potential high-use areas outside of the current array configuration.

Local Getis G*, a local indicator of spatial association (LISA), was useful in identifying areas of the receiver array with a disproportionate number of absences. G* is similar to other LISAs, such as Moran’s I, in that it identifies local clustering or spatial associations. A key difference that makes G* well-suited for acoustic telemetry applications is that high values of the G* statistic indicate hotspots and low values indicate coldspots, whereas high values of local Moran’s I indicate regions of high spatial autocorrelation (i.e., clusters of similar values, either high or low) [98]. It should be noted that a second local Getis statistic exists, G. G only considers the values at neighbouring locations and does not consider the value at the central location itself, whereas G* considers the values at neighbouring locations and the central location [73]. Acoustic telemetry studies are typically concerned with values at a specific location, so G* is likely more appropriate for most acoustic telemetry applications.

Local Getis G* indicated that areas immediately preceding or following absences or periods of non-detection were located along the coast, near the edge of the array, or at locations with larger distances to other receivers. The areas with high G* values could be prioritized for deployment of additional receivers to increase coverage and identify direction of travel during absences. Similarly, particularly for studies with limited resources, areas with low G* values (coldspots) could be further investigated to identify receivers that may be removed without unduly impacting results.

## Conclusions

We found that overwintering location was associated with date of freshwater return, providing the first evidence that length and/or difficulty affects migration timing of Arctic Char or Dolly Varden. Fish that had a longer and more challenging migration returned to freshwater earlier. Overwintering location within the Coppermine River system is known from a previous study to vary both among individuals and years (within individual) [22]. We found that movement or detection patterns in the marine environment also varied among individuals and years, but there was limited evidence that marine movement or detection patterns were associated with overwintering location. Although interannual variability in observed movement patterns suggests that species alone does not determine movement and space use in the marine environment, genetic analysis is required to ascertain the influence of species. A high proportion of absences or lack of detections in the array resulted in substantial uncertainty in some marine movement patterns and should be addressed in future research in this and other areas.

High-use locations, both in terms of movement and duration of detections, were largely similar between overwintering groups. Minor qualitative differences were observed in the proportion of time spent at key locations, likely due to differences in timing of freshwater return. Local (location-based) metrics revealed a preference for coastal locations for both overwintering groups, despite the array configuration likely biasing metrics towards offshore or island locations. Preference for coastal areas is likely due to the abundance of capelin observed in these areas, as well as patterns in break-up of sea ice. Similarities in space use between overwintering groups has implications for management and sustainability of the local fishery.

Although local indicators of spatial association have been used previously in aquatic acoustic telemetry [e.g., 40–42], their use remains uncommon. To our knowledge, we are the first to incorporate both local Getis G* and network analysis. Network analysis of spatial networks typically assesses the occurrence of movements between receivers [37, 38], whereas G*, as demonstrated here, is well-suited to assess the length of time spent at each receiver. If receivers have consistent and relatively high ranks using both local methods, it can be inferred with greater confidence that these locations are high-use locations. For these reasons, the two methods are complementary and, when used together, can provide a more comprehensive assessment of local spatial structure and space use in acoustic telemetry applications. We also demonstrated the usefulness of G* in identifying locations associated with absences or a lack of detections, which can be used to help focus future receiver deployments. We hope this approach will provide a useful example for future researchers.

### Electronic supplementary material

Below is the link to the electronic supplementary material.

Supplementary Material 1

## Data Availability

The datasets analysed during the current study are not publicly available due to their pertinence to the local Inuit fishery for Arctic Char and value to the community of Kugluktuk, but are available from the corresponding authors upon reasonable request.
