# Supplementary material for: Migration timing and marine space use of an anadromous Arctic fish (Arctic Char, Salvelinus alpinus) revealed by local spatial statistics and network analysis
Source: Mov Ecol. 2024 Feb 3;12:12. doi: 10.1186/s40462-024-00455-z (PMC10837978; doi:10.1186/s40462-024-00455-z)
Supplement: Supplementary file 1 — Supplementary Material 1 [file 40462_2024_455_MOESM1_ESM.docx]

Local Getis G* (hereafter, G*) was used to identify hotspots (clusters of high activity) and coldspots (clusters of low activity) in terms of time, rather than movement. G* values were calculated based on the duration of residence events at each receiver location. The threshold that was used to define residence events was 24 hours. The choice of threshold can influence both the number and duration of residence events. To assess whether the choice of residence threshold impacted the results and interpretation of G* values, the analysis was conducted using a range of thresholds (1, 2, 4, 6, 12, 18, 22, 23, 24, 25, 26, 36, and 48 hours). The G* values are presented in Fig. S1 and indicate that findings were consistent among residence thresholds.


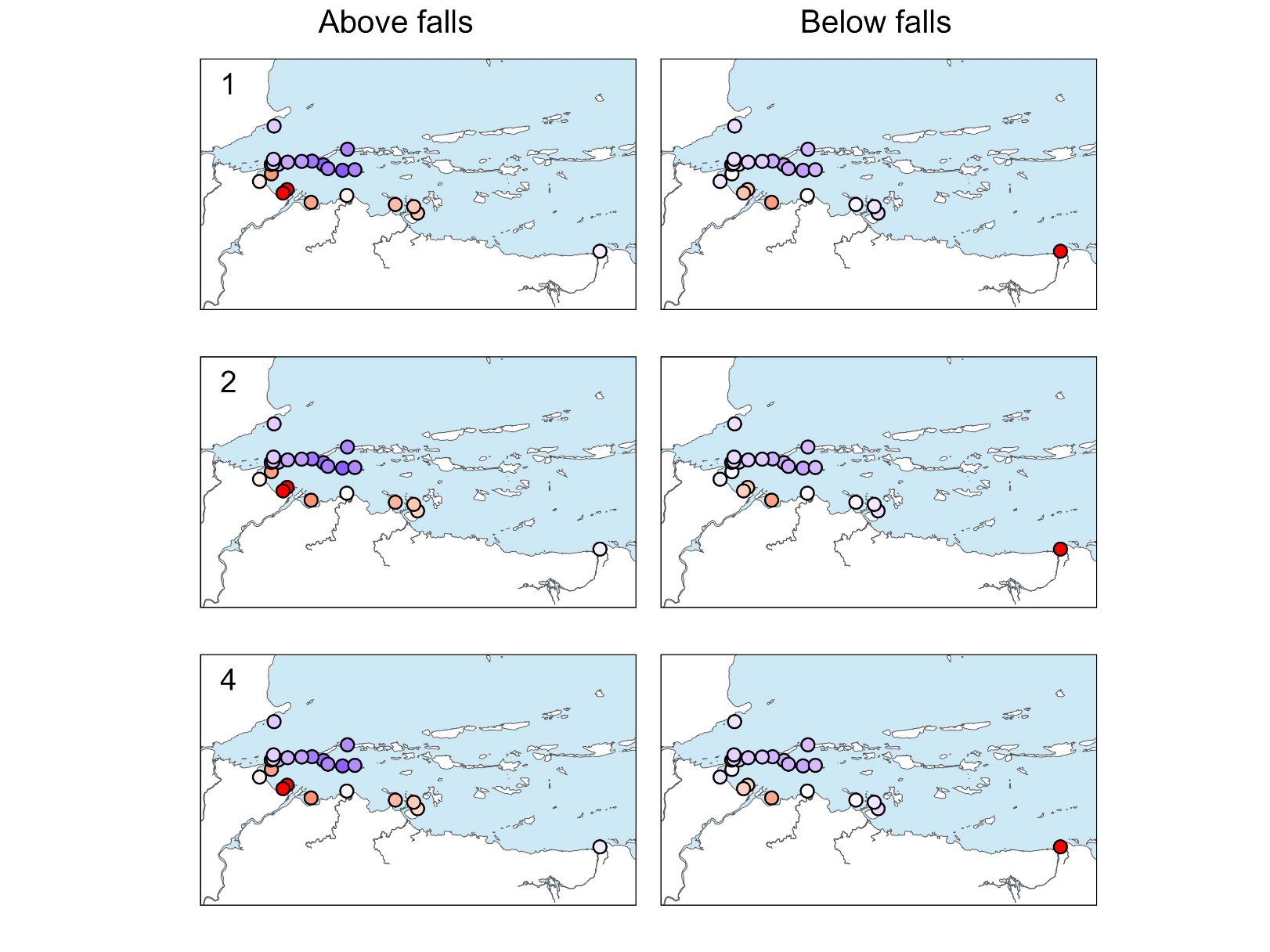


Fig. S1: Local Getis G* values for each receiver for fish that overwintered above Kugluk Falls (left panel) and fish that overwintered below Kugluk Falls (right panel). The residence threshold (hours) used to generate each plot is identified in the upper left corner of each row of plots. Red points represent receivers identified as hotspots, blue points represent receivers identified as coldspots, and white points represent G* values of 0, indicating no evidence of spatial association or hot/coldspots.


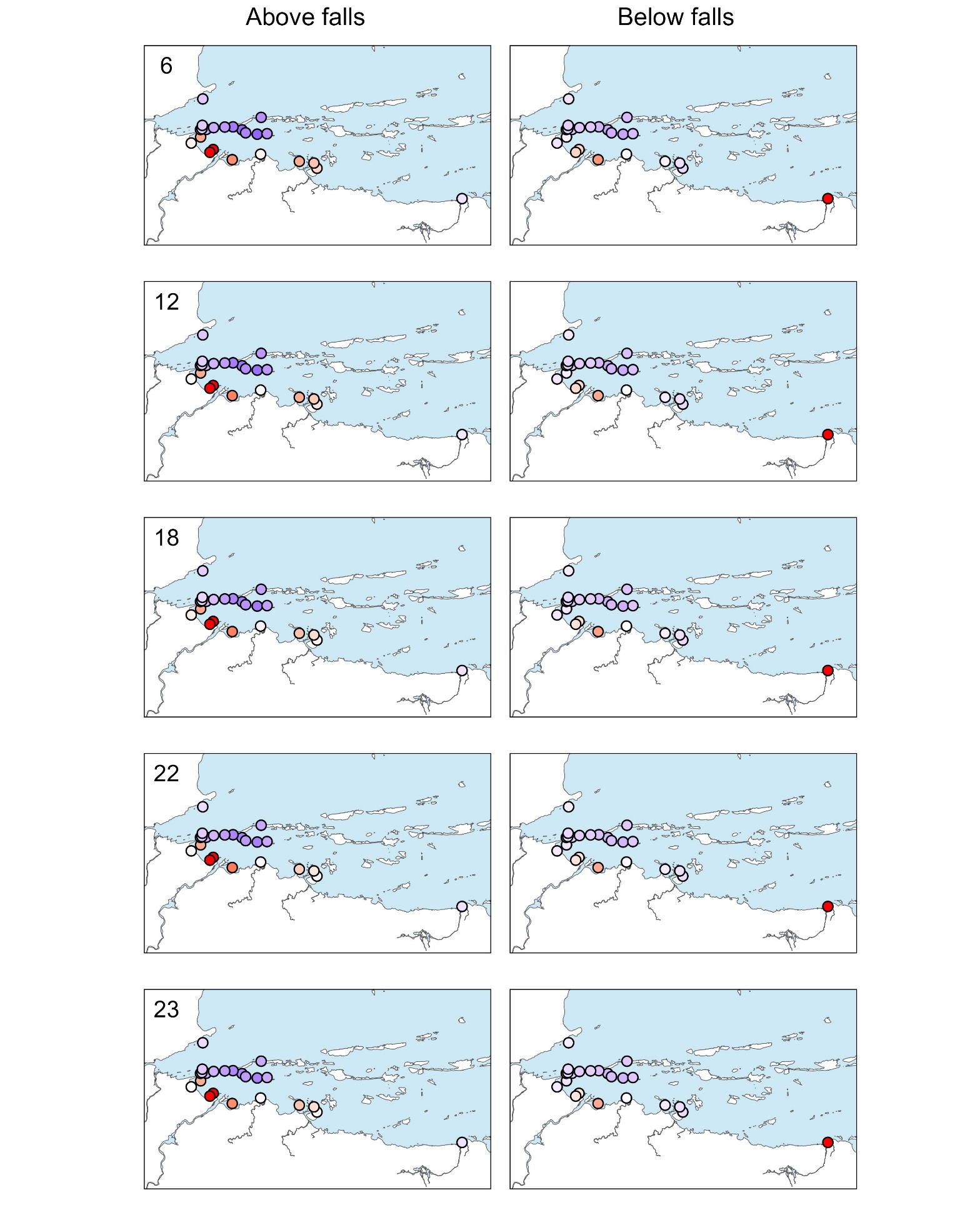


Fig. S1 continued


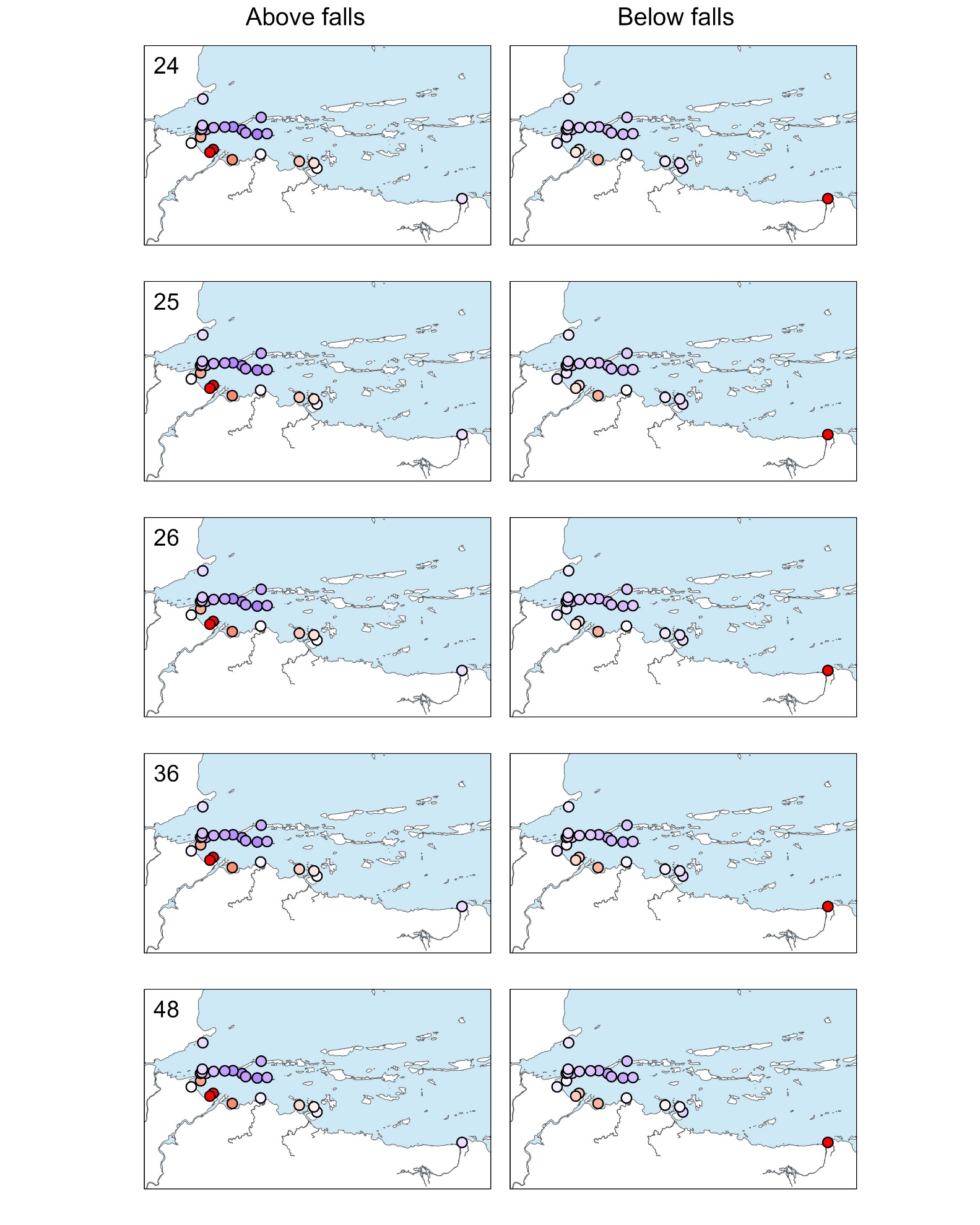


Fig. S1 continued
